# Leaf Spectra and Weight of Species in Canopy, Subcanopy, and Understory Layers in a Venezuelan Andean Cloud Forest

**DOI:** 10.6064/2012/839584

**Published:** 2012-05-23

**Authors:** Miguel F. Acevedo, Michele Ataroff

**Affiliations:** ^1^Department of Electrical Engineering, University of North Texas, Denton, TX 76203, USA; ^2^Department of Geography and Environmental Science Program, University of North Texas, Denton, TX 76203, USA; ^3^Centro de Simulación y Modelos (CESIMO), Universidad de Los Andes, Mérida 5101, Venezuela; ^4^Instituto de Ciencias Ambientales y Ecológicas (ICAE), Facultad de Ciencias, Universidad de Los Andes, Mérida 5101, Venezuela

## Abstract

We characterize the leaf spectra of tree species of an Andean cloud forest in Venezuela, grouped according to position in canopy, subcanopy and understory. We measured leaf reflectance and transmittance spectra in the 400–750 nm range using a high-resolution spectrometer. Both signals were subtracted from unity to calculate the absorbance signal. Nine spectral variables were calculated for each signal, three based on wide-bands and six based on features. We measured leaf mass per unit area of all species, and calculated efficiency of absorbance, as ratio of absorbance in photosynthetic range over leaf mass. Differences among groups were significant for several absorbance and transmittance variables, leaf mass, and efficiency of absorbance. The clearest differences are between canopy and understory species. There is strong correlation for at least one pair of band variables for each signal, and each band variable is strongly correlated with at least one feature variable for most signals. High canonical correlations are obtained between pairs of the three canonical axes for bands and the first three canonical axes for features. Absorbance variables produce species clusters having the closest correspondence to the species groups. Linear discriminant analysis shows that species groups can be sorted by all signals, particularly absorbance.

## 1. Introduction

Differences between understory and canopy environmental conditions are fundamental for forest dynamics. Among the many variables that distinguish both conditions, some are very important for physiological processes. In particular, light characteristics constitute a determinant factor in many biological processes [1–7].

Differences in solar radiation from the ground to the top of the canopy are determined by the optical properties of the leaves, that is absorption, reflection, and transmission of light [8, 9]. Leaves in different canopy strata have different optical properties, and these are related to ecological, physiological, biochemical, and anatomical characteristics [6, 10–21].

In particular, one leaf characteristic known to vary with canopy strata is specific leaf mass or leaf mass per unit area (LMA). In earlier studies, leaves of species in sunny versus shady environments at different locations showed no significant differences in absorbance, while showing differences in LMA [10]. Later results suggested differences in leaf spectral properties, as well as LMA, among species found in different positions of a vertical gradient (canopy, midcanopy, and understory) in a tropical forest [15]. Recent studies indicate that LMA variation is related to tree height rather than light conditions [22], that seasonal variations in leaf spectral properties are very high [23], and that physiological and morphological plasticity are essential for growth and reproduction in contrasting light environments [24].

Leaf reflectance spectra characteristics are key to understand the potential to distinguish species in remote sensing imagery [25–28] and to provide a tool for species identification [29]. All these efforts indicate that there are detectable differences in leaf spectral characteristics across species, and the environmental conditions in which they are immersed.

In cloud forests of tropical mountains, the elevated values of cloudiness and complex relief impose additional restrictions to the quantity and quality of solar radiation received in different vertical strata [2, 30, 31]. In this paper, we evaluate leaf spectral characteristics and LMA of twenty species, located in the canopy (15–25 m height), subcanopy (5–10 m), and understory (0–5 m) of an Andean cloud forest. We study the relationship of LMA and leaf spectra, and more importantly, we look for those leaf spectral characteristics that can best discriminate among the three groups of species.

We conducted this study in order to contribute data on leaf spectral properties of tropical cloud forests that could help support remote sensing species identification procedures, and to understand relationships between ecological processes and tree canopy position.

## 2. Methods

### 2.1. Study Area and Species

The study site is located at 2400 m elevation asl, 8°37′N and 71°10′W, in the Monterrey area, Valle Grande, near the city of Mérida, in the Andes of Venezuela. Mean annual temperature is 13°C and annual rainfall is 2560 mm with one peak in March–May and another in August–November. Distributed in several vertical strata, the vegetation is typical of upper montane Andean cloud forest and forms a relatively open canopy of 20–25 m of height [32].

We selected 20 species common in the Andean cloud forest (Table 1); of the nine canopy species, eight are trees, and the other, *Lycianthes ferruginea*, is a vine with leaves and reproductive parts always present in this upper strata; *Sapium stylare*, one of the three subcanopy species, was an individual of a canopy species that was still growing to reach this stratum, and therefore was considered to be in partial shade.

### 2.2. Experimental Setup

 For each species five mature leaves in good condition were collected and analyzed (09 January 2001). A few minutes elapsed between collection and measurement, well within the times for leaf spectra to remain unaltered by clipping; also, measurements focused on the visible range where spectra are unaltered by clipping [33, 34]. The leaves of canopy and subcanopy species were taken from a single individual, while the leaves of understory species were taken from five different individuals (because understory plants have fewer leaves).

In a dark room, we installed a black box with an orifice on one side where we placed a leaf to measure reflection and transmission of light coming from a lamp (ELH, 120 V, 300 W) located 178 cm from the leaf. The spectrum of the lamp in the range of interest (350–800 nm) was compared to the spectrum of solar radiation at the site. Even though the spectra were not exactly the same, the differences are compensated by taking the ratio of reflected and transmitted spectra to the spectrum of the lamp.

Two optical fibers (diameter 0.2 mm) conducted reflected and transmitted light from the leaf to a spectrometer. The fiber optic end to measure reflectance was placed at 2 cm from the leaf upper surface and the one for transmittance was placed at 2 cm of the lower surface of the leaf inside the black box. The fibers were connected to a portable fiber optic spectrometer (Ocean Optics SD-2000), which measures the spectrum between the ultraviolet (UV) and the near infrared (IR), in a range of 200–850 nm, using an array of 2048 diodes with an aperture slit of 100 *μ*m. The dispersion is (850 − 200)/2048 = 0.32 nm/diode, with a resolution of 12 × 0.32 = 3.8 nm (FWHM). For this study the signals were analyzed in a narrower range (400–750 nm) of interest in photosynthesis and plant responses to far red light. Data were taken simultaneously on two channels with identical specifications, using the two fibers; one to capture reflected light, and the other to measure transmitted light. In both cases, the fiber's end was bare which has a 25° field of view. The other end of each fiber was connected to an optical switch that allows blocking light to obtain dark response, which is subtracted from the signals in order to correct for electronic noise of the instrument.

In addition to leaf spectral characteristics, we determined LMA for five leaves of each species. For this purpose, we measured dry weight of the leaf blade and divided by the leaf area, which was measured using an LI-3100 Area Meter. Here we report LMA in mg/cm^2^.

### 2.3. Spectral Variables

 All spectral signals, reference (lamp) as well as reflected and transmitted, were smoothed by a seven-point central moving average [31]. Reflected and transmitted signals for the five leaves of each species were divided into the reference signal to obtain ratios of reflectance and transmittance as functions of wavelength *λ*. Both ratios were also smoothed by a seven-point central moving average to obtain reflectance *R*(*λ*) and transmittance *T*(*λ*) signals. Absorbance *A*(*λ*) for each leaf was then calculated as

(1) $$\begin{array}{c} A\left(\lambda \right)=1-R\left(\lambda \right)-T\left(\lambda \right). \end{array}$$

**Figure 1:**
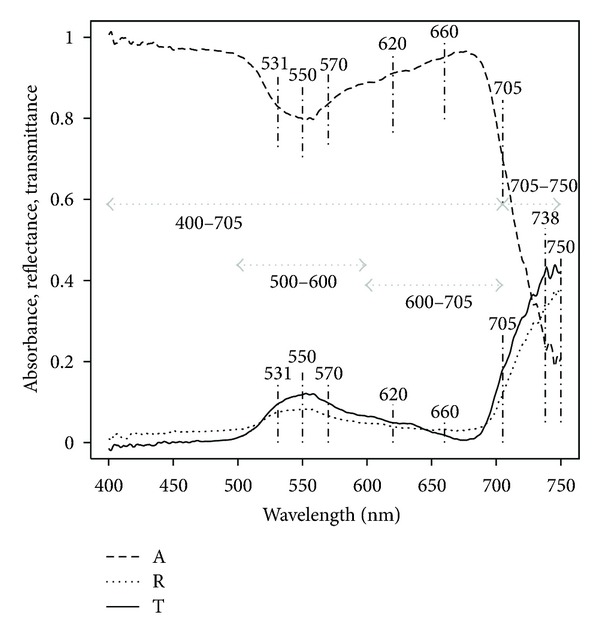
Bands and features selected for analysis. Shown for illustration are absorbance, reflectance, and transmittance spectra for one leaf of *Guettarda steyermarkii* (GS).

As an example, *A*(*λ*), *R*(*λ*), and *T*(*λ*) signals for one leaf of *Guettarda steyermarkii* (GS) are shown in Figure 1.

We defined a set of nine spectral variables for each signal (*A*, *R*, and *T*) as summarized in Table 2. We calculated the variables for each leaf and then averaged across leaves of the same species. Three of the nine variables correspond to averages over wide wavelength bands and the other six are features, that is, averages over a narrow band (Figure 1).

All variables are based on normalized differences and ratios reported in the literature; some of the variables have been proposed in reference to absorbance [10, 15, 20, 35], while others have been proposed for reflectance [19, 36, 37]. Instead of normalized difference we used a simple ratio because it yielded lower values of coefficient of variation across leaves. For brevity of presentation, the variables will be defined using the absorbance signal *A*(*λ*) only. However, the calculations also apply to the *T*(*λ*) and *R*(*λ*) signals except that we used the inverse of the ratio in order to obtain values less than 1 (Table 2).

For variables based on bands we will denote by *A*_*λ*_1_−*λ*_2__ the mean in the band *λ*_1_ − *λ*_2_ calculated as the integral of absorbance in this range divided by the bandwidth

(2) Aλ1−λ2=1λ2−λ1∫λ1λ2A(λ)dλ≅1λ2−λ1∑i=nλ1nλ2A(λi)×(λi−λi−1),  

where the integral is approximated by the sum of absorbance values multiplied by the interval between successive wavelengths. Here *n*_*λ*_*i*__ denotes the diode number for wavelength *λ*_*i*_.

Applying this equation, we calculate the mean absorbance in the photosynthetic range (400–705 nm) to define a first variable PHb,

(3) $$\begin{array}{c} \text{PHb}=A_{400–705}. \end{array}$$

In all calculations, we use 705 nm, the edge of chlorophyll absorption, instead of 700 nm [36–39].

Then, variable FRb, contribution of the spectrum in the far red (705–750 nm) relative to the one in the full photosynthetic range (400–705 nm), was calculated as the ratio of the integral in the first range (705–750 nm) over the integral in the second range (400–705 nm):

(4) $$\begin{array}{c} \text{FRb}=\frac{A_{700–750}}{A_{400–705}}. \end{array}$$

The third band variable is the ratio of the average spectrum in the green band relative to the red band:

(5) $$\begin{array}{c} \text{GRb}=\frac{A_{500-600}}{A_{600–705}}. \end{array}$$

When defining the next six variables, which are based on narrow bands or features, we will denote *Ai* as the absorbance at a particular wavelength calculated as the average over a narrow band (4 nm) around this wavelength; that is to say

(6) $$\begin{array}{c} A_{\lambda k}=\frac{\int_{\lambda k-2}^{\lambda k+2}A\left(\lambda \right)d\lambda }{\lambda k+2-\left(\lambda k-2\right)}≅\frac{\sum_{i=n_{\lambda k-2}}^{n_{\lambda k+2}}A\left(\lambda_{i}\right)\times \left(\lambda_{i}-\lambda_{i-1}\right)}{4} \end{array}$$

which was approximated by

(7) $$\begin{array}{c} A_{\lambda k}≅\frac{1}{N_{\lambda k}}\sum_{i=n_{\lambda k-2}}^{n_{\lambda k+2}}A\left(\lambda_{i}\right) \end{array}$$

because the wavelength differences were homogeneous over a small 4 nm interval. Here *N*_*λk*_ corresponds to the number of points in the 4 nm interval.

For example, absorbance *A*_620_ at 620 nm, wavelength at which maximum relative quantum efficiency occurs, is calculated using (7) in the interval from 618 to 622 nm. Our first feature variable MXf is simply *A*_620_:

(8) $$\begin{array}{c} \text{MXf}=A_{620}. \end{array}$$

Next, we define PDf, the magnitude of the depression in absorbance at 550 nm which is observed in all spectra (Figure 1). It was calculated as the ratio of average absorbance at 550 nm to the one at 660 nm:

(9) $$\begin{array}{c} \text{PDf}=\frac{A_{550}}{A_{660}}, \end{array}$$

where *A*_550_ and *A*_660_ are each calculated using (7).

Next, we use a concept similar to the Anthocyanin Reflectance Index which is a difference of the inverse of reflectance at 550 and 705 nm [19]. Instead of difference, we use a ratio of the value at 705 nm to the one at 550 nm. We will call it Anthocyanin Index (AIf):

(10) $$\begin{array}{c} \text{AIf}=\frac{A_{705}}{A_{550}}. \end{array}$$

For the next variable we use the concept of Photochemical Reflectance Index (PRI) based on a normalized index of reflectance at 570 and 531 nm; the first wavelength is a reference and the second corresponds with the xanthophyll pigment which in many plants relates with light use efficiency [36, 39–41]. Again instead of normalized difference we use a simple ratio and define it as photochemical index (PIf)

(11) $$\begin{array}{c} \text{PIf}=\frac{A_{531}}{A_{570}}. \end{array}$$

Based on the modified normalized difference vegetation index that uses the reflectance at 750 and 705 nm [36], and the simple ratio (SR) of reflectance at these wavelengths [42], we use a ratio of absorbance values at 705 and 750 nm to define a far-red Index (FRf):

(12) $$\begin{array}{c} \text{FRf}=\frac{A_{750}}{A_{705}}. \end{array}$$

In order to relate to the fluorescence peaks at 685 and 738 nm [36, 43, 44] we selected, as the next variable, the ratio of absorbance at 738 nm to the one at 570 nm (same reference used for PIf) as an absorbance index at the fluorescence peak (FIf):

(13) $$\begin{array}{c} \text{FIf}=\frac{A_{738}}{A_{570}}. \end{array}$$

The 738 nm feature was visually appreciable in the signals for canopy species but less so for subcanopy and understory. A variable related to the peak at 685 nm was explored but not used because the signals exhibited small differences among species and groups at this wavelength.

An additional composite variable, efficiency of absorbance per unit mass (EAM), combines a spectral variable PHb with LMA and was calculated as the ratio of *A*_400–705_ to the leaf weight LMA:

(14) $$\begin{array}{c} \text{EAM}=\frac{A_{400–705}}{\text{LMA}}=\frac{\text{PHb}}{\text{LMA}}. \end{array}$$

Its units are the inverse of LMA units; that is, are given here in cm^2^/mg. We used this ratio because historically vertical variations in LMA have been associated with light conditions [10, 15]. However, recent evidence indicates that vertical changes in LMA are more related to tree height [22].

### 2.4. Statistical Analysis

 Spectral variables were calculated using absorbance, reflectance, and transmittance for each leaf. Then, for each species the average, standard deviation, and coefficient of variation of all variables were calculated across the five leaves. We also calculated averages of leaf means and coefficient of variation for the three species groups, that is, canopy (C), subcanopy, (S) and understory (U). In addition, we calculated the leaf means and coefficient of variation of LMA and the leaf means of EAM.

The leaf means by species were used to conduct statistical tests and multivariate analysis among the species and among the groups. All tests and analyses were conducted separately for each signal absorbance, reflectance, and transmittance.

First, for each spectral variable we used a nonparametric analysis of variance (ANOVA, Kruskal-Wallis test) to detect differences among all three groups and a Wilcoxon test to compare each group pair. A multivariate analysis of variance (MANOVA, Wilks test) was used to determine if there were differences among the groups based on the full set of spectral variables. In addition, we conducted the same univariate tests (Kruskal-Wallis and Wilcoxon) for LMA and EAM. Then, we determined whether LMA can be predicted from the set of spectral variables by stepwise multiple linear regression.

Second, we conducted multivariate analysis procedures to examine the relationships among spectral variables, among species, and among groups of species. We started with the correlation matrix to study relationships between pairs of variables and conducted principal component analysis (PCA) to examine how many potential combinations of variables could account for most of the variance among species. Then, we conducted canonical correlation (CANCOR) analysis between the set of band variables and the set of feature variables to determine how well these sets explain each other. Next, we conducted hierarchical clustering using the Minkowski distance and the Ward method to examine relationships among species and compare clusters formed with the predefined groups. Finally, for the purpose of developing a linear combination of spectral variables that maximize differences among species groups we conducted a multiple linear discriminant analysis (LDA) using the spectral variables. All variables were standardized prior to the multivariate procedures described above.

Calculations and statistical analyses were programmed using the *R* system version 2.10.1 [45]. The program used to calculate the discriminant function is part of the MASS package for the *R* system [46].

## 3. Results

As exemplified in Figure 1, absorbance, reflectance and transmittance signals show expected patterns for all species. Notably, for reflectance and transmittance we see low values from 400 to 500 nm, a peak at ~550 nm, an elbow at ~690 nm, and a sharp increase in the 700–750 nm range (Figure 1). Such patterns produce a typical absorbance spectrum with high values from the beginning of the photosynthetic range, a valley at 550 nm, a recovery to high values at ~690 nm, and an abrupt drop to 750 nm (Figure 1).

**Figure 2:**
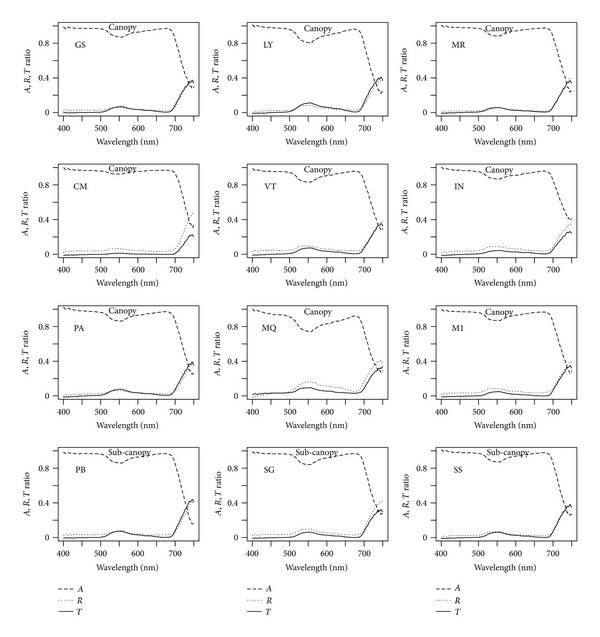
Absorbance (*A*), reflectance (*R*), and transmittance (*T*) spectra averaged across leaves for canopy and subcanopy species.

**Figure 3:**
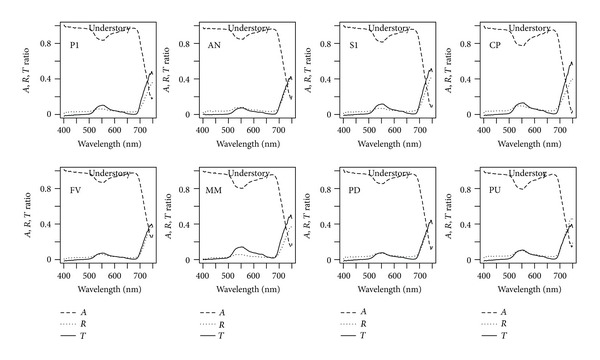
Absorbance (*A*), reflectance (*R*), and transmittance (*T*) spectra averaged across leaves for understory species.

Absorbance values at the 550 nm depression, at the recovery past 660 nm, and the far red decline from 705 to 750 nm showed variations among canopy species and understory species (Figures 2 and 3), but less so among subcanopy species (Figure 2). The most contrasting canopy, species are CM and MQ with little and strong reduction at 550 nm respectively (Figure 2). In the understory group, CP and PU showed the most important reduction at 550 nm, while FV, PD, and AN, showed the least important reduction at 550 nm (Figure 3). Similar patterns are noted for transmittance; however, reflectance spectra are very similar among species and groups (Figures 2 and 3).

**Figure 4:**
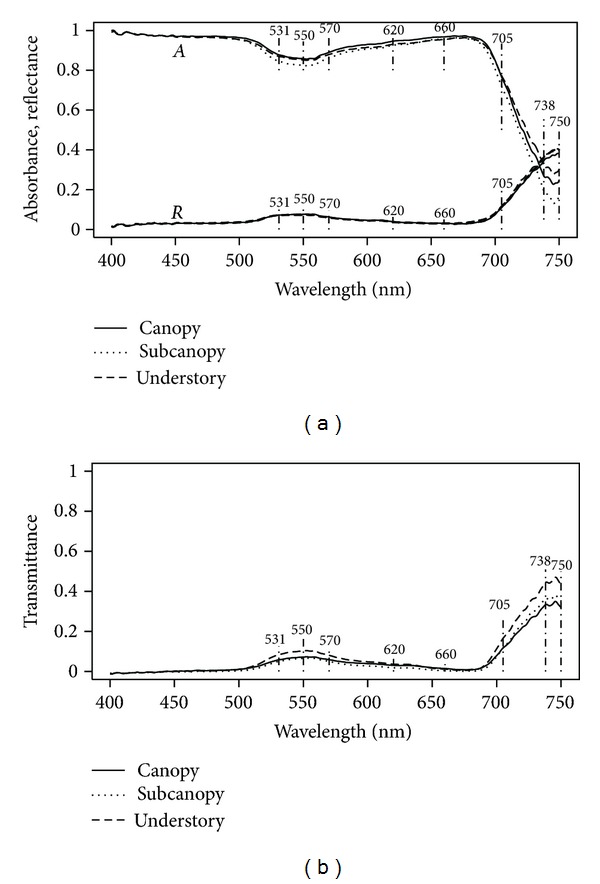
Spectra for each group (canopy, subcanopy, and understory) averaged across species. (a) Absorbance (*A*) and Reflectance (*R*). (b) Transmittance (*T*).

Spectra averaged by group showed differences in absorbance and transmittance among groups, but very small differences in reflectance (Figure 4). Particularly, the 550 nm feature and the far red slope show clear differences among groups of species for absorbance and transmittance, and small differences for reflectance. In addition, notable at 550 nm the absorbance is less for understory species, followed by canopy species and then slightly larger for subcanopy, even though these last two groups have similar values (Figure 4). At 660 nm the largest absorbance corresponds to subcanopy, being understory and canopy lower and similar to each other (Figure 4). Between 570 and 620 nm the absorbance shows the clearest difference among the groups, being larger for subcanopy, followed by canopy, and then by understory. From 700 to 750 nm the differences increase with wavelength and show a gradient from understory, to subcanopy, and canopy. In addition, two small valleys, one at 738 nm and another in between 738 and 750 nm are noticeable in all groups. The patterns just described for absorbance are also apparent in the transmittance signal but of course inverted in sign. The double valley (peaks in this case) in 738–750 nm is more accentuated (Figure 4).

Group average and standard deviation of the coefficients of variation (CV) across leaves are given in Table 3. There is relatively low variability (~1-2%) across leaves for all absorbance variables except for far red features (FRf, FIf) which have high CV values (~15–30%), and the far red band FRb which has intermediate variability (~8-9%). There is high variability (~15–45%) across leaves for reflectance and transmittance except for PIf which is in the range ~2-3% and GRb which is ~5–8%.

**Figure 5:**
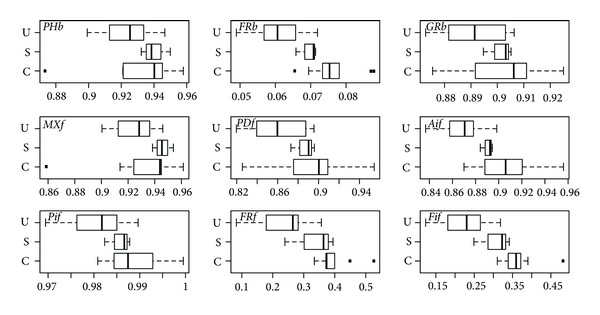
Leaf means of absorbance variables by group (canopy C, subcanopy S, and understory U).

**Figure 6:**
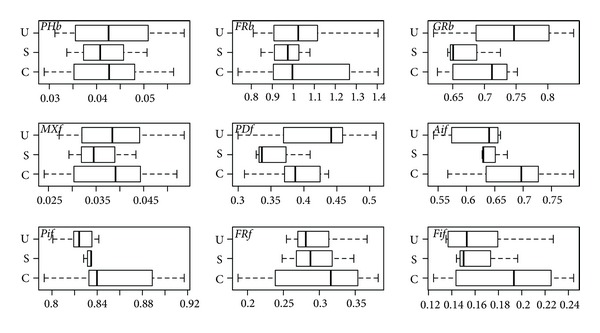
Leaf means of reflectance variables by group (canopy C, subcanopy S, and understory U).

**Figure 7:**
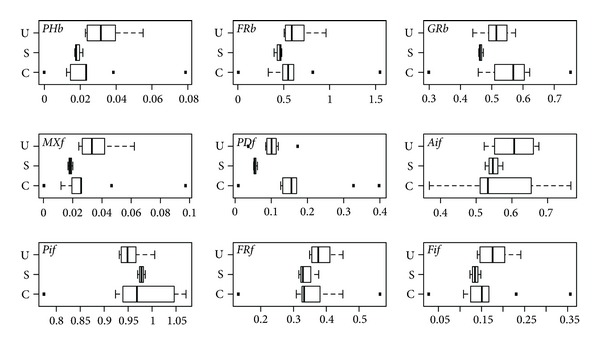
Leaf means of transmittance variables by group (canopy C, subcanopy S, and understory U).

Leaf means of all spectral variables suggest patterns in the group differences and within-group variability. For absorbance (Figure 5), potentially significant differences among groups are not evident except for FRb, AIf, FRf, and FIf. Differences in group averages of all variables are very small for reflectance; moreover, the variability within groups is large for all variables (Figure 6). Potentially significant differences in group averages (and with lower within-group variability) are more evident in a few transmittance variables; PHb, FRb, and MXf (Figure 7). However, there is frequent occurrence of extreme values for canopy species in most variables.

There are no significant (*P* < 5%) differences among all three groups or pairs of groups in reflectance (Table 4) except between canopy and understory species for AIf. Differences among all three groups (Table 4) were found to be significant in absorbance for FRb, AIf, FRf, and FIf, and in transmittance for PHb, MXf, and PDf. This suggests the importance of far red absorbance and the photosynthetic-range transmittance in separating groups. Subcanopy species had significant differences with understory species only. Even though these two groups exhibit significant differences only in MXf absorbance, they have significant differences in transmittance forPHb, FRb, MXf, and FIf.

The clearest differences are between canopy and understory species. These groups show significant differences in absorbance for FRb, PIf, FRf, and FIf (all related to far red except for PIf), in reflectance for AIf, and in transmittance for PDf (Table 4). None of the signals are significantly different among groups according to a MANOVA (Wilks test) using all spectral variables (*P* ~ 0.06, 0.33, 0.11 for absorbance, reflectance, and transmittance, resp.).

Weight and the composite variable (LMA and EAM) exhibit differences among groups (Table 5). Group average of LMA is highest for canopy, followed by understory and lowest for subcanopy. Variability across leaves is relatively high, with CV being highest for canopy, followed by subcanopy, and being lowest for understory. Significant differences are found among all groups (Kruskal-Wallis *P*-value 0.023 and 0.01 for LMA and EAM respectively) and between canopy and understory for both LMA (*P* = 0.021) and EAM, (*P* = 0.008), and between canopy and subcanopy for EAM (*P* = 0.036).

Stepwise regression analysis of LMA as a function of spectral variables generated the best predictor based on all variables (except PHb) for absorbance, on four variables (MXf, AIf, PIf, and FIf) for reflectance, and on only two variables (FRb, and PDf) for transmittance. The adjusted *R* ^2^ for these regressions are 0.64, 0.52, and 0.90 for absorbance, reflectance and transmittance, respectively. The *P*-values indicate a very significant trend in all cases, and particularly for transmittance (0.007, 0.004, and 1.7 × 10^−9^ respectively). The *P*-values for the coefficients of the regression for transmittance are very significant (~4  × 10^−9^). However, all coefficients for absorbance have *P*-values exceeding 5%, and those for reflectance are in the 1–5% range. CM, having a LMA much larger than any other species generates a very large 5% confidence interval at high values of LMA.

Correlation analysis (Table 6) shows strong correlation coefficients (>0.85) for at least one pair of band variables for each signal: GRb-FRb in absorbance, PHb-FRb in both reflectance and transmittance. Also, one feature variable FIf is correlated with other feature variables: with FRf for absorbance, with MXf and FRf in reflectance, and with MXf, FRf, and AIf in transmittance. Each band variable is strongly correlated with at least one feature variable for all signals, except GRb in transmittance. The pair PHb-MXf has strong correlation for all signals, whereas PIf show low correlation values with other variables for all signals.

Three principal components explain more than 95% of the variance for absorbance and transmittance, and nearly 90% for reflectance (Table 7, PCA). Very high canonical correlations are obtained between pairs of the three canonical axes for bands and the first three canonical axes for features (Table 7, CANCOR). The first pair of axes have correlation values larger than 0.995, the second pair of axes has values larger than ~0.95, and the third pair has values larger than 0.89. The correlations are always highest for absorbance (Table 7, CANCOR). The square of the SVD (singular value decomposition) terms obtained by the LDA and their ratios show much higher discrimination power for the first axis (LD1) compared to the second (LD2) for absorbance (~3.5*x*) and reflectance (~8*x*), but nearly the same for transmittance (~1.1*x*) (Table 7, LDA).

**Figure 8:**
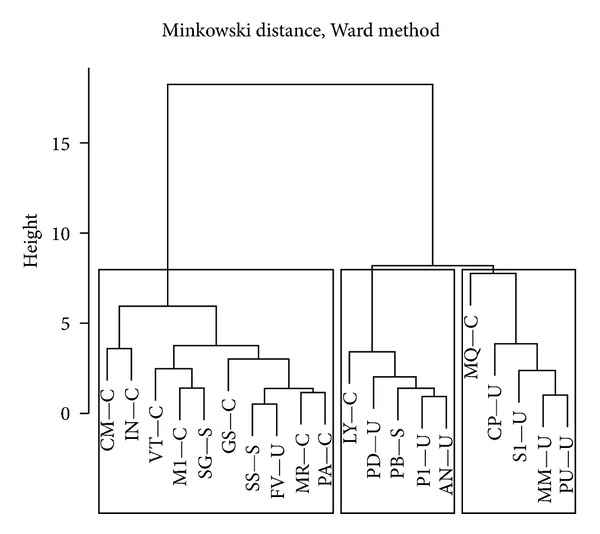
Dendrogram obtained from cluster analysis using absorbance. Branches are labeled by species and groups (canopy C, subcanopy S, and understory U). It was cut at a distance (height) such that it generates three clusters; these are shown by rectangular boxes.

Dendrograms from the cluster analysis were cut at a distance (height) generating three clusters. Of all signals, absorbance produces species clusters having the closest correspondence to the pre-defined species groups (Figure 8). Cluster 1 (leftmost box) consists mostly of canopy species and two of the subcanopy species (SS and SG); whereas clusters 2 and 3 (middle and rightmost boxes) are mostly understory species with a couple of canopy species (LY and MQ) and the other subcanopy species (PB). The only understory species included in cluster 1 is FV, which joins SS at low height. However, two canopy species are included in clusters 2 and 3; LY in cluster 2, and MQ in cluster 3. Both join these clusters at higher nodes and thus are relatively dissimilar to all the understory species.

**Figure 9:**
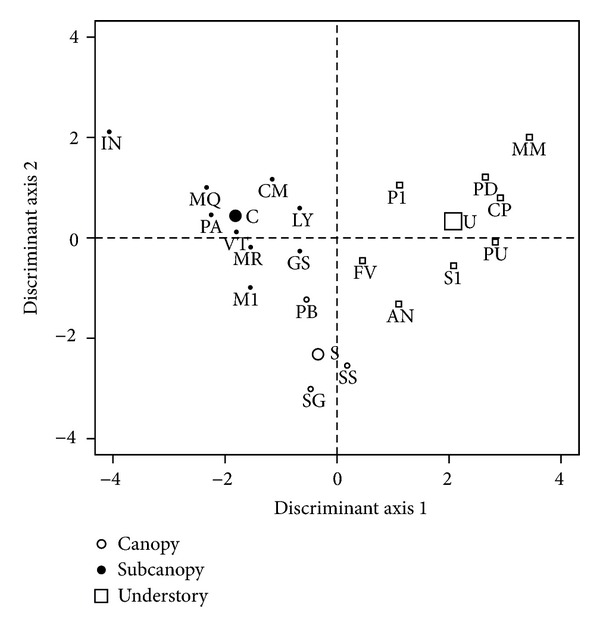
Discriminant space obtained by LDA using absorbance variables. Shown are centroid (large symbols) and species locations (small symbols) for the three groups.

LDA results are similar for all three signals. As an example Figure 9 shows the results for absorbance. We can clearly appreciate differences among groups. The first axis (LD1) discriminates between canopy and understory species. Subcanopy species are located at intermediate positions of this axis, but separated from the other two groups by the second axis (LD2). All canopy species are in the negative part of LD1 whereas understory species have positive values. IN is at the extreme of canopy species, whereas PD and MM are at the extreme of understory species (Figure 9). For the sake of space we do not include LDA coefficients but we observed that with relatively higher values GRb, PDf, FRf, and FIf contribute to LD1, while FRb, GRb, PDf, and FIf contribute the most to LD2.

## 4. Discussion

Absorbance, reflectance, and transmittance spectra display patterns similar to the ones reported for other tropical forests [10, 15, 20]. Absorbance spectra showed variations among species in the 550 nm depression and the far red decline (Figures 2 and 3). These differences are noticeable for canopy and understory species, but less so for subcanopy species. Absorbance in the 500–705 nm range is less for understory species, followed by canopy species and subcanopy (Figures 2 and 3). This is evident at 550 nm confirming previous results in rain forests [15]. From 705 nm the largest absorbance corresponds to canopy species, which is a different result for three of the four species measured at La Selva, Costa Rica [15].

Canopy species presented higher values of LMA, compared to subcanopy and understory species. This result confirms vertical differences of LMA observed in all forests [22]. Our LMA values are in the same range as those reported for La Selva [15] but lower than those reported for a cloud forest in Puerto Rico [20]. This finding may suggest less severe light or water restrictions in our site when compared to the cloud forest in Puerto Rico. There is substantial variability in LMA as indicated by relatively large values ~10–17% of CV (Table 5). LMA differences are significant only when comparing canopy and understory species; however there are significant differences in EAM between canopy and subcanopy as well.

Several spectral variables show differences among the groups of species, mostly those variables related to far red and weight (Table 4). The only others are MXf, showing differences between subcanopy and understory, and PIf which shows differences between canopy and understory (Table 4). In general, the difference between subcanopy and canopy groups and between subcanopy and understory are less appreciable than the differences between canopy and understory species (Table 4).

For absorbance, variables related to far red, FRb, FRf, and FIf all display a gradient from high to low for canopy, subcanopy and understory leaves (Figure 5). For FRf and FIf this is due to a lesser absorbance decline from 705 to 750 nm for subcanopy and canopy when compared to understory (Figure 4). For FRb however, the pattern is an indication of increasing absorbance at 550 nm from understory to canopy and subcanopy.

EAM allows differentiation among canopy and subcanopy groups, and canopy and understory, but not between subcanopy and understory. EAM is lower for canopy species, which is due to larger values of LMA, thus confirming previous results in other forests [10, 15]. Historically, this finding is interpreted in terms of lower plant's cost to produce the leaf mass needed to achieve required absorbance. However, recent evidence indicates that vertical changes in LMA are due to tree height because of different water restrictions at higher canopy levels [22].

It is interesting that only two transmittance variables explain 90% of LMA. The regression coefficients indicate that estimated LMA decreases with FRb and increases with PDf. This finding would suggest that leaves with higher mass will transmit less light and thus suppress growth in lower forest strata. However, upon further scrutiny of this result we found that this relation may not be robust because of high leverage by CM. We ran the regression analysis again after removing CM from the data set. The adjusted *R* ^2^ for the best predictors declined to 0.38, 0.30, and 0.60 for absorbance, reflectance, and transmittance, respectively. Furthermore, four variables are required by the best predictor based on transmittance and only two (GRb and MXf) had *P*-values < 0.05.

Strong correlation between PHb with MXf, indicates the importance of 620 nm in explaining absorbance over the photosynthetic range. Similarly, the strong correlation between PDf with GRb, indicates that the depression at 550 nm (relative to the mid of the red-band) explains most of the difference between the red and green bands. Other two strong correlations correspond to the variables in the far red, FRb with FIf, and FRf with FIf.

Three principal components suffice to account for more than 90% of the variance suggesting co-linearity among many variables. Our approach was to select variables based on ratios of well-known bands and features. Variables could be selected by pattern recognition methods and other features may be found. For example, when separating leaves of trees and lianas in tropical dry forests, based on reflectance spectra, as many as 10–100 features are selected [47].

Absorbance variables can generate one species cluster related to canopy species and two other clusters related to understory species (Figure 8). These two clusters join at a slightly higher distance and can be considered as one cluster related mostly to understory species. However, subcanopy species do not form a separate cluster, but mix within the other clusters; SS and SG with the canopy cluster and PB with the understory clusters. This finding confirms that species differences are much more marked between canopy and understory, and that subcanopy tends to be similar to canopy. It should be noted that we treated SS as a subcanopy species because leaves were taken from an individual in midcanopy, but normally trees of this species reach the canopy.

As shown by the LDA, the first axis separates species along a gradient from canopy to understory (left to right in Figure 9). Satisfyingly, subcanopy species are intermediate along this axis. Of the three subcanopy species, SS has positive values and is closest with understory species, particularly to FV (also suggested by the cluster analysis). The other two, SG and PB, have negative values and are closest to the canopy species, especially to GS and LY (but recall that PB came closest to understory in the cluster analysis).

Only one LDA axis would suffice except for slight differences of subcanopy species with extremes of the other two groups. Further separation of subcanopy species with respect to the other two groups is provided by the second LDA axis. However, PB remains close to other groups along this axis as well. In this case, M1 (canopy group) and AN (understory group) have a position approaching the subcanopy group. There is relatively good separation among species themselves along the two axes; although we did not address differentiation at the species level, our results indicate that it may be feasible.

## Figures and Tables

**Table 1:** List of species studied in canopy, subcanopy, and understory of the cloud forest in Monterrey, Estado Mérida, Venezuela.

Species	Code	Family	Life form
Canopy (15–25 m)			
* Guettarda steyermarkii* Standl.	GS	Rubiaceae	Tree
* Lycianthes ferruginea* Bitter	LY	Solanaceae	Vine
* Miconia resimoides* Cogn.	MR	Melastomataceae	Tree
* Clusia multiflora* H.B.K.	CM	Clusiaceae	Tree
* Viburnum tinoides* L.f.	VT	Caprifoliaceae	Tree
* Inga* sp.	IN	Mimosaceae	Tree
* Piper aduncum* DC	PA	Piperaceae	Tree
* Montanoa quadrangularis* Sch.Bip.	MQ	Asteraceae	Tree
* Miconia* sp.	M1	Melastomataceae	Tree
Subcanopy (5–10 m)			
* Piper bogotense* DC	PB	Piperaceae	Tree
* Solanum gratum* Bitter	SG	Solanaceae	Tree
* Sapium stylare* Müll.Arg.	SS	Euphorbiaceae	Tree
Understory (0–5 m)			
* Psychotria* sp.	P1	Rubiaceae	Shrub
* Anthurium nymphaeifolium* Koch & Bouché	AN	Araceae	Herbaceous
* Solanum* sp.	S1	Solanaceae	Shrub
* Chamaedorea pinnatifrons* (Jacq.) Oerst.	CP	Arecaceae	Palm
* Fuchsia venusta* H.B.K.	FV	Onagraceae	Shrub
* Miconia meridensis* Triana	MM	Melastomataceae	Shrub
* Palicourea demissa* Standl.	PD	Rubiaceae	Shrub
* Psychotria aubletiana* Steyerm.	PU	Rubiaceae	Herbaceous

**Table 2:** Summary of variables selected for analysis.

Type	Code	Description	Calculation		
			Absorb.	Reflec.	Transm.
	PHb	Average in photosynthetic range	*A*_400–705_	*R*_400–705_	*T*_400–705_
Bands	FRb	Average in far red relative to average in photosynthetic range	*A*_705–750_/*A*_400–705_	*R*_400–705_/*R*_705–750_	*T*_400–705_/*T*_705–750_
	GRb	Average in green relative to average in red	*A*_500–600_/*A*_600–705_	*R*_600–705_/*R*_500–600_	*T*_600–705_/*T*_500–600_

	MXf	Value at maximum relative quantum efficiency	*A*_620_	*R*_620_	*T*_620_
	PDf	Green feature	*A*_550_/*A*_660_	*R*_660_/*R*_550_	*T*_660_/*T*_550_
Features	AIf	Anthocyanin index	*A*_705_/*A*_550_	*R*_550_/*R*_705_	*T*_550_/*T*_705_
	PIf	Photochemical index	*A*_531_/*A*_570_	*R*_570_/*R*_531_	*T*_570_/*T*_531_
	FRf	Far red index	*A*_750_/*A*_705_	*R*_705_/*R*_750_	*T*_705_/*T*_750_
	FIf	Far red fluorescence peak	*A*_738_/*A*_570_	*R*_570_/*R*_738_	*T*_570_/*T*_738_

Weight	LMA	Leaf weight per unit area in mg/cm^2^	Dry weight divided into leaf area		

Composite	EAM	Efficiency of absorbance (in cm^2^/mg)	*A*_400–705_/LMA		

**Table 3:** Group average ± standard deviation of coefficient of variation (CV, in %) of leaf means. Groups (Gp.) are canopy (C), sub-canopy (S), and understory (U). Spectra (Sp.): absorbance (*A*), reflectance (*R*), and transmittance (*T*).

Sp.	Gp.	PHb	FRb	GRb	MXf	PDf	AIf	PIf	FRf	FIf
	C	1.58 ± 1.05	8.86 ± 2.67	0.88 ± 0.33	1.60 ± 1.06	2.10 ± 0.86	2.14 ± 1.64	0.47 ± 0.25	15.89 ± 6.24	14.36 ± 6.26
*A*	S	1.89 ± 1.14	9.63 ± 3.62	0.93 ± 0.15	1.88 ± 0.98	2.29 ± 0.49	2.26 ± 1.07	0.25 ± 0.05	17.28 ± 4.99	17.23 ± 4.49
	U	1.88 ± 0.96	8.28 ± 3.20	0.96 ± 0.39	1.87 ± 0.86	2.12 ± 0.97	1.52 ± 0.54	0.38 ± 0.20	30.40 ± 12.62	22.22 ± 8.93

	C	26.21 ± 17.06	21.33 ± 16.07	8.37 ± 5.07	26.33 ± 17.25	20.28 ± 13.79	8.43 ± 6.14	3.59 ± 2.26	11.99 ± 7.82	15.79 ± 12.19
*R*	S	39.13 ± 21.41	32.96 ± 17.15	8.17 ± 1.08	44.67 ± 22.30	23.26 ± 7.90	10.89 ± 6.08	3.64 ± 1.67	15.52 ± 2.76	29.73 ± 12.31
	U	34.16 ± 21.01	29.38 ± 19.36	8.04 ± 6.22	36.62 ± 22.82	22.40 ± 20.46	8.30 ± 4.28	2.72 ± 1.13	12.85 ± 5.78	22.98 ± 11.77

	C	33.74 ± 10.72	20.74 ± 8.78	6.53 ± 5.99	35.70 ± 14.65	26.44 ± 22.97	7.74 ± 3.98	2.61 ± 2.07	12.59 ± 8.48	18.27 ± 8.03
*T*	S	30.52 ± 12.19	20.22 ± 4.28	6.71 ± 3.33	33.98 ± 10.37	39.77 ± 20.05	5.11 ± 2.34	1.52 ± 0.31	10.56 ± 2.35	16.52 ± 1.85
	U	22.77 ± 12.54	15.81 ± 8.79	5.29 ± 2.95	24.85 ± 14.70	28.36 ± 27.29	6.46 ± 3.97	1.86 ± 0.87	9.00 ± 4.38	13.50 ± 7.11

**Table 4:** Significance (*P*-values in %) of differences among all groups (C-S-U) by Kruskal-Wallis test. and between groups by Wilcoxon test (*P*-values <5% are highlighted in bold). Groups are canopy (C), sub-canopy (S), and understory (U).

Spectra	Groups compared	PHb	FRb	GRb	MXf	PDf	AIf	PIf	FRf	FIf
	All	28.10	**0.32**	22.40	15.60	10.20	**1.95**	7.09	**0.41**	**0.14**
*A*	C-S	100.00	14.50	72.70	48.20	48.20	60.00	60.00	37.30	10.00
	S-U	13.30	13.30	37.60	**4.85**	19.40	8.48	27.90	19.40	8.48
	C-U	27.70	**0.06**	11.40	32.10	5.92	**0.79**	**2.74**	**0.03**	**0.02**

	All	95.48	77.80	24.00	83.70	27.46	7.47	12.60	93.33	73.79
*R*	C-S	86.36	60.00	60.00	60.00	37.27	20.91	28.18	86.36	100.00
	S-U	92.12	63.03	27.88	77.58	19.39	63.03	37.58	92.12	77.58
	C-U	88.84	88.84	16.72	96.26	37.04	**3.60**	7.45	81.48	48.07

	All	**4.57**	7.41	15.77	**3.64**	**2.26**	46.02	29.59	18.94	12.74
*T*	C-S	60.00	20.91	20.91	37.27	**6.36**	100.00	86.36	72.73	48.18
	S-U	**1.21**	**1.21**	8.48	**1.21**	8.48	27.88	13.33	19.39	**4.85**
	C-U	9.27	42.34	32.13	9.27	**3.60**	37.04	32.13	13.88	23.59

**Table 5:** LMA by species, group average for LMA and EAM, and coefficient of variation (CV, in %) across leaves for LMA.

Canopy		Subcanopy		Understory	
Species	LMA (mg/cm^2^)	Species	LMA (mg/cm^2^)	Species	LMA (mg/cm^2^)
GS	14.86 ± 2.28	PB	3.20 ± 0.21	P1	4.85 ± 0.51
LY	5.4 ± 1.86	SG	4.35 ± 0.28	AN	6.49 ± 0.83
MR	9.46 ± 1.59	SS	5.42 ± 1.65	S1	4.64 ± 0.45
CM	30.43 ± 2.16			CP	4.72 ± 0.17
VT	9.43 ± 2.2			FV	6.66 ± 0.95
IN	10.13 ± 1.6			MM	4.68 ± 0.51
PA	7.97 ± 1.33			PD	5.48 ± 0.48
MQ	4.67 ± 0.89			PU	3.87 ± 0.18
M1	5.28 ± 0.30				

Group average ± Std Dev					
	Canopy	Subcanopy		Understory	

LMA (mg/cm^2^)	10.85 ± 7.99	4.32 ± 1.11		5.17 ± 0.97	
LMA leaf CV (%)	17.13 ± 8.52	14.48 ± 13.82		9.39 ± 3.68	
EAM (cm^2^/mg)	0.12 ± 0.05	0.23 ± 0.06		0.18 ± 0.03	

**Table 6:** Correlation between spectral variables for all spectra (absorbance *A*, reflectance *R*, and transmittance *T*). High values (>0.85) are highlighted in bold. Italics denote high correlation between bands or between features. Underlined cells show correlations of FIf with other feature variables.

Spec		FRb	GRb	MXf	PDf	AIf	PIf	FRf	FIf
*A*	PHb	0.52	***0.88***	**0.98**	**0.88**	0.49	0.23	0.18	0.32
	FRb		0.71	0.44	0.77	**0.87**	0.57	**0.86**	**0.95**
	GRb			0.83	**0.99**	0.60	0.45	0.38	0.53
	FIf			0 .22	0.58	0.74	0.65	***0.97***	

*R*	PHb	***0.90***	−0.03	**0.98**	0.05	0.52	0.14	0.65	0.80
	FRb		0.02	**0.94**	0.12	0.62	0.27	0.69	**0.90**
	GRb			0.05	**0.91**	−0.49	0.06	−0.16	−0.23
	FIf			***0.85***	−0.20	0.61	0.43	***0.89***	

*T*	PHb	***0.97***	0.68	**0.99**	0.42	**0.86**	0.36	**0.88**	**0.96**
	FRb		0.79	**0.98**	0.44	**0.88**	0.51	**0.92**	**0.99**
	GRb			0.72	0.37	0.58	0.78	0.77	0.74
	FIf			***0.96***	0.36	***0.91***	0.52	***0.95***	

**Table 7:** PCA: Proportion of accumulated variance of the first three principal components. CANCOR: correlations between canonical axes. LDA: Singular value decomposition (SVD) terms and ratios.

PCA		Comp. 1	Comp. 2	Comp. 3
	*A*	0.646	0.881	0.952
Accumulated variance	*R*	0.536	0.770	0.889
	*T*	0.740	0.877	0.966

CANCOR		Can. 1	Can. 2	Can. 3

	*A*	1.000	0.996	0.973
Correlation coefficient	*R*	0.995	0.987	0.968
	*T*	0.999	0.949	0.890

LDA		LD1	LD2	LD1/LD2

	*A*	31.188	8.998	3.466
SVD square	*R*	22.961	2.879	7.975
	*T*	15.880	14.397	1.103

## References

[1] Young JE, Evans GC, Bainbridge R, Rackham O (1975). Effects of the spectral composition of light sources on the growth of a higher plant. *Light as an Ecological Factor: II*.

[2] Huber O (1978). Light compensation point of vascular plants of a tropical cloud forest and an ecological interpretation. *Photosynthetica*.

[3] Smith H, Briggs WR, Green PB, Jones RL (1982). Light quality, photoperception, and plant strategy. *Annual Review of Plant Physiology*.

[4] Lee DW (1987). The spectral distribution of radiation in two neotropical rainforests. *Biotropica*.

[5] Endler JA (1993). The color of light in forests and its implications. *Ecological Monographs*.

[6] Lee DW, Baskaran K, Mansor M, Mohamad H, Yap SK (1996). Irradiance and spectral quality affect asian tropical rain forest tree seedling development. *Ecology*.

[7] Grant RH (1997). Partitioning of biologically active radiation in plant canopies. *International Journal of Biometeorology*.

[8] de Castro F (2000). Light spectral composition in a tropical forest: measurements and model. *Tree Physiology*.

[9] Giuliani R, Brown KJ (2008). Within-canopy sampling of global irradiance to describe downwelling light distribution and infer canopy stratification in a broadleaf forest. *Tree Physiology*.

[10] Lee DW, Graham R (1986). Leaf optical properties of rainforest sun and extreme shade plants. *American Journal of Botany*.

[11] Lee DW, Bone RA, Tarsis SL, Storch D (1990). Correlates of leaf optical properties in tropical forest sun and extreme-shade plants. *American Journal of Botany*.

[12] St-Jacques C, Labrecque M, Bellefleur P (1991). Plasticity of leaf absorbance in some Broadleaf tree seedlings. *Botanical Gazette*.

[13] Carter GA (1993). Responses of leaf spectral reflectance to plant stress. *American Journal of Botany*.

[14] Wright SJ, van Schaik CP (1994). Light and the phenology of tropical trees. *American Naturalist*.

[15] Poorter L, Oberbauer SF, Clark DB (1995). Leaf optical properties along a vertical gradient in a tropical rain forest canopy in Costa Rica. *American Journal of Botany*.

[16] Baldini E, Facini O, Nerozzi F, Rossi F, Rotondi A (1997). Leaf characteristics and optical properties of different woody species. *Trees-Structure and Function*.

[17] Ávalos G, Mulkey SS, Kitajima K (1999). Leaf optical properties of trees and lianas in the outer canopy of a tropical dry forest. *Biotropica*.

[18] Lee DW, Oberbauer SF, Johnson P (2000). Effects of irradiance and spectral quality on leaf structure and function in seedlings of two Southeast Asian Hopea (dipterocarpaceae) species. *American Journal of Botany*.

[19] Gitelson AA, Merzlyak MN, Chivkunova OB (2001). Optical properties and nondestructive estimation of anthocyanin content in plant leaves. *Photochemistry and Photobiology*.

[20] Cordero RA, Fetcher N (2002). *Absorbancia Foliar de 19 Especies de un Bosque Nuboso Enano en Puerto Rico*.

[21] Souza RP, Válio IFM (2003). Leaf optical properties as affected by shade in saplings of six tropical tree species differing in successional status. *Brasilian Journal of Plant Physiology*.

[22] Cavaleri MA, Oberbauer SF, Clark DB, Clark DA, Ryan MG (2010). Height is more important than light in determining leaf morphology in a tropical forest. *Ecology*.

[23] Hesketh M, Sánchez-Azofeifa GA (2012). The effect of seasonal spectral variation on species classification in the Panamanian tropical forest. *Remote Sensing of Environment*.

[24] Letts MG, Rodríguez-Calcerrada J, Rolo V, Rambal S (2012). Long-term physiological and morphological acclimation by the evergreen shrub Buxus sempervirens L. to understory and canopy gap light intensities. *Trees-Structure and Function*.

[25] Castro-Esau KL, Sánchez-Azofeifa GA, Caelli T (2004). Discrimination of lianas and trees with leaf-level hyperspectral data. *Remote Sensing of Environment*.

[26] Clark ML, Roberts DA, Clark DB (2005). Hyperspectral discrimination of tropical rain forest tree species at leaf to crown scales. *Remote Sensing of Environment*.

[27] Castro-Esau KL, Sánchez-Azofeifa GA, Rivard B, Wright SJ, Quesada M (2006). Variability in leaf optical properties of mesoamerican trees and the potential for species classification. *American Journal of Botany*.

[28] Zhang J, Rivard B, Sánchez-Azofeifa A, Castro-Esau K (2006). Intra- and inter-class spectral variability of tropical tree species at La Selva, Costa Rica: implications for species identification using HYDICE imagery. *Remote Sensing of Environment*.

[29] da Luz BR (2006). Attenuated total reflectance spectroscopy of plant leaves: a tool for ecological and botanical studies. *New Phytologist*.

[30] Acevedo M, Monteleone S, Ataroff M, Estrada C (2001). Aberturas del dosel y espectro de la luz en el sotobosque de una selva nublada andina de Venezuela. *Ciencia*.

[31] Acevedo M, Ataroff M, Monteleone S, Estrada C (2003). Heterogeneidad estructural y lumínica del sotobosque de una selva nublada andina de Venezuela. *Interciencia*.

[32] Ataroff M, Kappelle M, Brown AD (2001). Venezuela. *Bosques Nublados del Neotrópico*.

[33] Foley S, Rivard B, Sanchez-Azofeifa GA, Calvo J (2006). Foliar spectral properties following leaf clipping and implications for handling techniques. *Remote Sensing of Environment*.

[34] Thomasson JA, Sui R (2009). Cotton leaf reflectance changes after removal from the plant. *Journal of Cotton Science*.

[35] Osborne BA, Raven JA (1986). Light absorption by plants and its implication for photosynthesis. *Biological Reviews*.

[36] Gamon JA, Surfus JS (1999). Assessing leaf pigment content and activity with a reflectometer. *New Phytologist*.

[37] Gitelson AA, Gritz Y, Merzlyak MN (2003). Relationships between leaf chlorophyll content and spectral reflectance and algorithms for non-destructive chlorophyll assessment in higher plant leaves. *Journal of Plant Physiology*.

[38] Gitelson A, Merzlyak MN (1994). Quantitative estimation of chlorophyll-a using reflectance spectra: experiments with autumn chestnut and maple leaves. *Journal of Photochemistry and Photobiology B*.

[39] Gamon JA, Kitajima K, Mulkey SS, Serrano L, Wright SJ (2005). Diverse optical and photosynthetic properties in a neotropical dry forest during the dry season: implications for remote estimation of photosynthesis. *Biotropica*.

[40] Peñuelas J, Filella L (1998). Technical focus: visible and near-infrared reflectance techniques for diagnosing plant physiological status. *Trends in Plant Science*.

[41] Garrity SR, Vierling LA, Bickford K (2010). A simple filtered photodiode instrument for continuous measurement of narrowband NDVI and PRI over vegetated canopies. *Agricultural and Forest Meteorology*.

[42] Castro-Esau KL, Sánchez-Azofeifa GA, Rivard B (2006). Comparison of spectral indices obtained using multiple spectroradiometers. *Remote Sensing of Environment*.

[43] Meroni M, Colombo R (2006). Leaf level detection of solar induced chlorophyll fluorescence by means of a subnanometer resolution spectroradiometer. *Remote Sensing of Environment*.

[44] Meroni M, Rossini M, Guanter L (2009). Remote sensing of solar-induced chlorophyll fluorescence: review of methods and applications. *Remote Sensing of Environment*.

[45] CRAN

[46] Venables WN, Ripley BD (2002). *Modern Applied Statistics with S*.

[47] Kalacska M, Bohlman S, Sanchez-Azofeifa GA, Castro-Esau K, Caelli T (2007). Hyperspectral discrimination of tropical dry forest lianas and trees: comparative data reduction approaches at the leaf and canopy levels. *Remote Sensing of Environment*.

